# The 150 most important questions in cancer research and clinical oncology series: questions 67–75: Edited by *Chinese Journal of Cancer*

**DOI:** 10.1186/s40880-017-0254-z

**Published:** 2017-11-01

**Authors:** Chinese Journal of Cancer

**Affiliations:** Sun Yat-sen University Cancer Center, Guangzhou, 510060 Guangdong P. R. China

**Keywords:** Resistant to chemotherapy, Hepatocellular carcinoma, RAS, Cancer-targeted therapy, Oligomer, Non-invasive early detection and diagnosis, Lung cancer, Volatolome, Heavy ion beam radiotherapy, Targeted alpha-particle therapy, Chromosomal instability

## Abstract

Since the beginning of 2017, *Chinese Journal of Cancer* has published a series of important questions in cancer research and clinical oncology, which sparkle diverse thoughts, interesting communications, and potential collaborations among researchers all over the world. In this article, 9 more questions are presented as followed. Question 67. How could we overcome the resistance of hepatocellular carcinoma against chemotherapeutics? Question 68. Is pursuit of non-covalent small-molecule binders of RAS proteins viable as a strategy of cancer drug discovery? Question 69. In what oligomeric structures do RAS proteins signal? Question 70. How can we achieve non-invasive early detection and diagnosis of lung cancer? Question 71. Does genetic information influence the volatolome enabling diagnosis of lung cancer with genetic mutations via cell headspace or breath analysis? Question 72. Is heavy ion beam radiotherapy effective to kill cancer stem cells? Question 73. Is there any diversity among different types of cancer in terms of sensitivity to heavy ion beam radiotherapy? Question 74. Can targeted alpha-particle therapy augment the effect of carbon ion radiotherapy on malignancies? Question 75. How does chromosomal instability drive tumor progression?

## Text

To accelerate our endeavors to overcome cancer, *Chinese Journal of Cancer* has launched a program of publishing 150 most important questions in cancer research and clinical oncology [1]. Since the beginning of 2017, *Chinese Journal of Cancer* has published a series of important questions in cancer research and clinical oncology [2–10], which sparkle diverse thoughts, interesting communications, and potential collaborations among researchers all over the world. In this article, Questions 67–75 are selected and presented. This program of collecting and publishing the key questions is still ongoing. Please send your thoughtful questions to Ms. Ji Ruan via email: ruanji@sysucc.org.cn.

## Question 67: How could we overcome the resistance of hepatocellular carcinoma against chemotherapeutics?

### Background and implication

Hepatocellular carcinoma (HCC) is the sixth most common cancer and the second leading cause of cancer-related deaths worldwide. Over 80% of HCCs are diagnosed at advanced stages, indicating that only a few patients are eligible for radical surgery, radiofrequency ablation, or liver transplantation. Sorafenib is the only first-line systemic therapeutic drug approved by the USA Food and Drug Administration for advanced HCC over the last 10 years. Although regorafenib has been approved at the end of 2016 as the second-line therapy for sorafenib-resistant cases, the survival benefit of patients has been far below expectation.

Compared with other digestive tract solid tumors, HCC is very resistant against cytotoxic drugs with overexpression of several important proteins responsible of detoxification, including dihydropyrimidine dehydrogenase and multidrug-resistance proteins. In addition, patients with advanced HCC usually have notable underlying liver disease, which is associated with poor tolerability to systemic chemotherapy.

The difficulties we have faced in the systemic therapy for HCC indicate that inducing the sensitivity of HCC cells to chemotherapy is an important issue for promoting therapeutic outcome of the patients. The interaction between HCC cells and endothelial cells might be a key element for establishing the defense system of HCC, and therefore targeting the vasculature of HCC might be helpful for improving its sensitivity toward cytotoxic drugs.

#### Submitter

Zhongguo (Roy) Zhou.

#### Affiliation and email

Sun Yat-sen University Cancer Center, Guangzhou, Guangdong 510060, P. R. China.

zhouzhg@sysucc.org.cn.

## Question 68: Is pursuit of non-covalent small-molecule binders of RAS proteins viable as a strategy of cancer drug discovery?

### Background and implication

RAS proteins play central roles in cell signaling that regulates the growth, proliferation, and differentiation of cells. It is such an important drug target that the former director of National Institute of Health and Nobel laureate Harold Varmus wrote that “If we had such a weapon [a RAS drug], a large fraction of the most lethal human cancers would suddenly become much more amenable to treatment.” While small-molecule covalent inhibitors have been developed, this strategy is limited to G12C RAS mutant. After years of trying, it remains a pending question whether non-covalent inhibitors of desirable pharmacological properties suitable for orally available drugs are viable to target wild-type RAS proteins and other RAS mutants that are prevalent in cancer patients. Clearly, a novel screening and drug design technology should be invented to identify the effective small molecules targeting wild-type RAS as well as mutant RAS. Achievement in this direction will open a new door for cancer-targeted therapy.

#### Submitter

Yibing Shan.

#### Affiliation and email

D. E. Shaw Research, New York, NY 10036, USA.

Beijing Computational Science Research Center, Beijing 100193, P. R. China.

Yibing.shan@deshawresearch.com.

## Question 69: In what oligomeric structures do RAS proteins signal?

### Background and implication

RAS proteins are crucial signaling proteins coupled with receptor tyrosine kinases in regulating cell growth, proliferation, and differentiation. Against the conventional wisdom of the field, more and more data indicate that RAS proteins, like many other signaling proteins, form complex high-order oligomers that contains scaffold proteins and downstream effectors on the cytoplasmic membrane upon it activation. The oligomers serve as potent signaling platforms yielding threshold responses to the input signal, and their formation involves a phase-transition-like process that is highly sensitive to the local environment and to concentrations, localizations, post-translational modifications, and mutations of the proteins involved. The mechanisms by which these factors control the formation, disassembly, and the temporal behavior of RAS oligomers, and especially the structure of such signaling oligomers of RAS remain the central questions in RAS study. Answering these questions would lead to new strategies for drug discovery targeting RAS signaling.

#### Submitter

Yibing Shan.

#### Affiliations and email

D. E. Shaw Research, New York, NY 10036, USA.

Beijing Computational Science Research Center, Beijing 100193, P. R. China.

Yibing.shan@deshawresearch.com.

## Question 70: How can we achieve non-invasive early detection and diagnosis of lung cancer?

### Background and implication

Early detection of lung malignancy is integral for increasing the survival rates of the patients. Extensive work is being done on the development of a non-invasive diagnostic test for lung cancer that analyzes volatile organic compounds (VOCs) via exhaled breath [11]. In recent years, there is growing evidence that the underlying metabolic process related to the development and progression of cancer leads to the release of VOCs from cells to the blood and out in the exhaled breath [12]. The analysis of these biomarkers is referred to as volatolomics. A major risk factor for cancer development is associated with increased oxidative stress and the induction of cytochrome p450 enzymes [12]. In the body, oxidative stress is connected to the overall equilibrium between the creation and deactivation of reactive oxygen species (ROS) and free radicals. Other sources of ROS could be from exogenous origins, for example, cigarette smoke, pollution, and radiation [13]. Once accumulated in the tissue, ROS can attack different molecules in the body such as polyunsaturated fatty acids and proteins generating VOCs that are emitted in the breath [13]. Therefore, breath analysis using different mass-spectrometric techniques or sensor-based systems is currently in development and shows great promise in worldwide studies. The sensor technology, as artificially intelligent nanoarray based on molecularly modified gold nanoparticles, is probably the most promising approach, due to its fast and relatively cheap point of care potential, for diagnostics and the monitoring of lung cancer treatment [14, 15].

#### Submitter

Hossam Haick.

#### Affiliation and email

Department of Chemical Engineering and Russell Berrie Nanotechnology Institute, Technion-Israel Institute of Technology, Haifa 3200003, Israel.

hhossam@technion.ac.il.

## Question 71: Does genetic information influence the volatolome enabling diagnosis of lung cancer with genetic mutations via cell headspace or breath analysis?

### Background and implication

Volatolome is the collective data representing metabolite volatile organic compounds (VOCs) in the body [16]. This approach is being used for disease detection and diagnostics. Lung cancer is one such disease that is eligible for breath analysis. Gene expression profiling is gaining importance in accurately classifying tumors and personalizing treatment for individual patients [17]. Genetic alterations associated with tumor growth can lead to volatolomic changes in the intracellular microenvironment [16]. Metabolic changes occur all the time in normal and abnormal processes in the body. Genetic information is normally phenotypically expressed in the production of different metabolites as VOCs. Genetic information changes due to DNA damage can result in a null product, an altered product, or occasionally, a change in the concentration of a VOC product [16]. For lung cancer and other malignancies, subcategories of the disease are characterized by specific genetic mutations [17, 18]. Therefore, screening for genetic mutations is currently the best option for personalizing treatment. Volatolomic in vitro studies of a number of lung cancer-related mutations, including Epidermal Growth Factor Receptor (EGFR), Kirsten rat sarcoma viral oncogene (KRAS), and activin-like kinase EML4 (EML4-ALK), and tumor protein *p53 (TO53)* have shown promising results in distinguishing mutant versus wild-type cells using nanomaterial-based sensor systems [17, 18]. Such results may lead to VOC-based diagnostics and genetic mutation profiling detection from the headspace of a lung cancer tissue specimen obtained through biopsy or from patients’ breath [17–19].

#### Submitter

Hossam Haick.

#### Affiliation and email

Department of Chemical Engineering and Russell Berrie Nanotechnology Institute, Technion-Israel Institute of Technology, Haifa 3200003, Israel.

hhossam@technion.ac.il.

## Question 72: Is heavy ion beam radiotherapy effective to kill cancer stem cells?

### Background and implication

Cancer cells can be killed by unrepairable DNA double-strand breaks. Photon radiation-induced DNA damage is mainly single-strand break and therefore sublethal. Accumulation of sublethal DNA single-strand breaks also contributes to lethality. There are several radioprotective mechanisms for cancer stem cells to repair sublethal damage, resulting in more resistance in cancer stem cells to radiotherapy in contrast to non-stem cancer cells [20]. Heavy ion beam radiotherapy has several advantages in comparison to traditional photon radiotherapy. The first one is the sharp dose fall-off behind the Bragg peak (BP), allowing very good protection to the surrounding normal tissues. The second advantage is to induce much more DNA double-strand breaks in cancer cells and consequently making DNA repair much more inefficient [21]. The third advantage is elevated relative biological effect (RBE) and less dependent on oxygen enhancement ratio (OER), which is calculated via the following formula: OER = radiation dose in hypoxia/radiation dose in the air. With these multiple unique advantages, it is therefore reasonable to hypothesize that heavy ion beam radiotherapy can be more effective in killing cancer stem cells. Evidence in animal and cell line models has been collected to support this hypothesis. Clinical evidence of the effective tumor control in human patients has also been accumulated worldwide including Japan, Germany, Italy, Austria, and China [22]. It is more promising if the heavy ion beam radiotherapy could be combined with radiosensitizing microRNAs targeting cancer stem cells [23].

#### Submitter

Masayuki Kumada.

#### Affiliation and email

Department of Accelerator and Medical Physics, National Institute of Radiological Science, 4-9-1 Anagawa, Inage-ku, Chiba-shi, Chiba 263-8555, Japan.

infofeynman@gmail.com.

## Question 73: Is there any diversity among different types of cancer in terms of sensitivity to heavy ion beam radiotherapy?

### Background and implication

The advantages of heavy ion radiotherapy include the drop of radiation dose to almost zero beyond the Bragg peak, and generation of much more DNA double-strand damage in contrast to photon or proton radiation. Carbon ion (heavier than proton and often regarded as heavy ion, sometimes called light ion) beam radiotherapy has been shown to be effective in treating melanoma [24], lung cancer [25], liver cancer [26], colorectal cancer [27], head and neck cancer [28], and kidney cancer [29]. Based on the experience of Yamagata University Hospital, the diseases suitable for heavy ion therapy include the tumors of the head and neck, pulmonary and mediastinal, gastrointestinal tract (esophagus, rectum, recurrent colon inside the pelvis), liver, gallbladder, pancreas, urologic tract (prostate and kidney), breast, and bone and soft tissues, as well as locally advanced gynecologic cancers (locally advanced uterine cervical cancer, locally advanced cervical endometrial cancer, malignant melanoma of the gynecologic area) and metastatic cancers (metastatic lung tumor, metastatic liver cancer, metastatic lymph nodes) [30].

However, there is no report on the sensitivity of solid tumors to heavy ion beam radiotherapy. Study of what cancer types are more sensitive or more resistant to heavy ion beam radiotherapy could be beneficial for further improving the treatment outcome by heavy ion radiotherapy. Randomized clinical trials may be helpful to clarify this issue [31].

#### Submitter

Masayuki Kumada.

#### Affiliation and email

Department of Accelerator and Medical Physics, National Institute of Radiological Science, 4-9-1 Anagawa, Inage-ku, Chiba-shi, Chiba 263-8555, Japan.

infofeynman@gmail.com.

## Question 74: Can targeted alpha-particle therapy augment the effect of carbon ion radiotherapy on malignancies?

### Background and implication

Alpha particles release short-ranged high-energy radiations capable of killing 1–3 cells. We attempted to deliver alpha-emitting isotopes to leukemia cells and small solid tumors using antibodies as ligands [32]. Bi-213 and Ac-225 are purified and chelated to the antibodies. Biochemistry of the agents, radiobiology, and pharmacology have been studied. Disease model systems under study include myeloid leukemia, prostate cancer, and lymphoma, all with promising results. It is therefore believed that targeted alpha-particle therapy (TAT) can augment the effect of carbon ion radiotherapy on non-solid cancer or microscopic metastatic lesions. Clinical trials will be initiated once the clinical scale-up is accomplished. The challenging issue is the limited availability of Bi-213 and Ac-225, which can be generated from thorium molten salt reactor (MSR). There is a hope that new MSRs might be built in China and India.

#### Submitter

Masayuki Kumada.

#### Affiliation and email

Department of Accelerator and Medical Physics, National Institute of Radiological Science, 4-9-1 Anagawa, Inage-ku, Chiba-shi, Chiba 263-8555, Japan.

infofeynman@gmail.com.

## Question 75: How does chromosomal instability drive tumor progression? Background and application

### Background and implication

Chromosomal instability (CIN), defined by an elevated rate of chromosome mis-segregation and breakage, results in diverse chromosomal aberrations in tumor cell populations. Accumulating cytogenetic analyses of over 60,000 cases of human cancer have indicated that most solid tumors contain chromosomal aberrations, with each tumor displaying a distinct abnormal karyotype (Mitelman database: http://cgap.nci.nih.gov/Chromosomes/Mitelman). In typical human cancers, one-quarter of the genome is affected by arm-level copy number aberrations. Cancer genome sequencing has revealed dynamic chromosomal content changes during clonal evolution of the tumor cell population. However, how chromosomal loss or gain drives tumor progression to metastasis remains unknown. It is technically difficult to determine the biological function of a specific chromosomal content change, which may influence the expression of hundreds to thousands of genes. Recently, advanced genome-editing techniques have been used to delete large chromosomal region, even whole chromosomal arm [33, 34]. With the application of new methodology, the findings on chromosomal content changes in continuingly isolated phenotypic variants of tumor cells might shed some light on the role of CIN in driving tumor metastatic phenotypic switching [35]. These studies have proved the concept that CIN is playing an important role in cancer progression. Since CIN is one of the most common features of cancer cells, it is believed that CIN could be a potential therapeutic target.

#### Submitter

Chong-Feng Gao.

#### Affiliation and email

Van Andel Research Institute, Grand Rapids, Michigan 49503, USA.

Chongfeng.gao@vai.org.
